# Radiopacity of Sodium Zirconium Cyclosilicate in Computed Tomography: a case of a patient with Hyperkalemia and kidney disease

**DOI:** 10.1080/0886022X.2023.2284839

**Published:** 2023-11-20

**Authors:** Andrea Angioi, Matteo Floris, Nicola Lepori, Gianfranca Cabiddu, Antonello Pani

**Affiliations:** a Nephrology, Dialysis and Transplantation Unit, ‘Giuseppe Brotzu’ Hospital, Cagliari, Italy; b Department of Medical Science and Public Health, University of Cagliari, Cagliari, Italy

**Keywords:** Sodium Zirconium Cyclosilicate, radiopacity, Computed Tomography (CT) imaging, hyperkalemia, Chronic Kidney Disease (CKD), diagnostic imaging

## Abstract

Sodium Zirconium Cyclosilicate (SZC) is commonly used for treating hyperkalemia because it sequesters gastrointestinal potassium ions, thereby reducing serum potassium levels. However, a less-discussed aspect of SZC is its radiopacity on x-ray-based imaging techniques. The European Medicines Agency (EMA) has only vaguely addressed this issue. Radiopaque substances like SZC can interfere with diagnostic imaging, creating challenges for clinicians and radiologists. We present the case of a 34-year-old Italian male to illustrate these concerns.

We draw attention to a little-discussed yet clinically relevant issue concerning Sodium Zirconium Cyclosilicate (SZC) for treating hyperkalemia, specifically in kidney failure. Hyperkalemia, defined as serum potassium levels exceeding 5.0 mEq/L, is associated with considerable morbidity and mortality risks, especially cardiac arrhythmias. This is particularly alarming for patients with kidney failure, with prevalence rates ranging from 14% to 20%, stratified according to the KDIGO classification 2012 [1].

While SZC effectively sequesters gastrointestinal potassium ions, reducing serum potassium levels [2], emerging data suggests its radiopacity on x-ray-based imaging modalities. The European Medicines Agency (EMA) has provided only a circumspect suggestion on this issue [3]. Radiopaque substances can compromise diagnostic accuracy, posing challenges for clinicians and radiologists [4].

We describe the case of a 34-year-old Italian male with a history of untreated hypertension and recent hospitalization (May 2023) for nephrotic-nephritic syndrome associated with previously unrecognized kidney failure (proteinuria: 7.6 g/24 h, hypoalbuminemia: 2.7 g/dL, serum creatinine: 3 mg/dL, active urinary sediment). He tested negative for autoimmune conditions during his initial hospitalization, including ANA, ENA screening, anti-dsDNA, ANCA, and anti-GBM. A kidney biopsy was not conducted due to severe anemia and thrombocytopenia. After discharge, the patient continued outpatient follow-up care.

By July 2023, he was re-hospitalized for an elective ultrasound-guided kidney biopsy after an improved platelet count. The biopsy, devoid of intra or post-procedural complications, presented preliminary results indicative of an immunocomplex mediated membranoproliferative glomerulonephritis. Concurrent ultrasonography exposed hitherto undetected splenomegaly (longitudinal diameter: 18 cm). Subsequent imaging, a whole-body CT scan with and without intravenous contrast, unveiled no splenic focal lesions. However, an unanticipated radiopacity in the small intestine and colon was evident in the non-contrast scan, as if oral contrast had been administered before imaging (Figures 1 (A–C).

**Figure 1.**
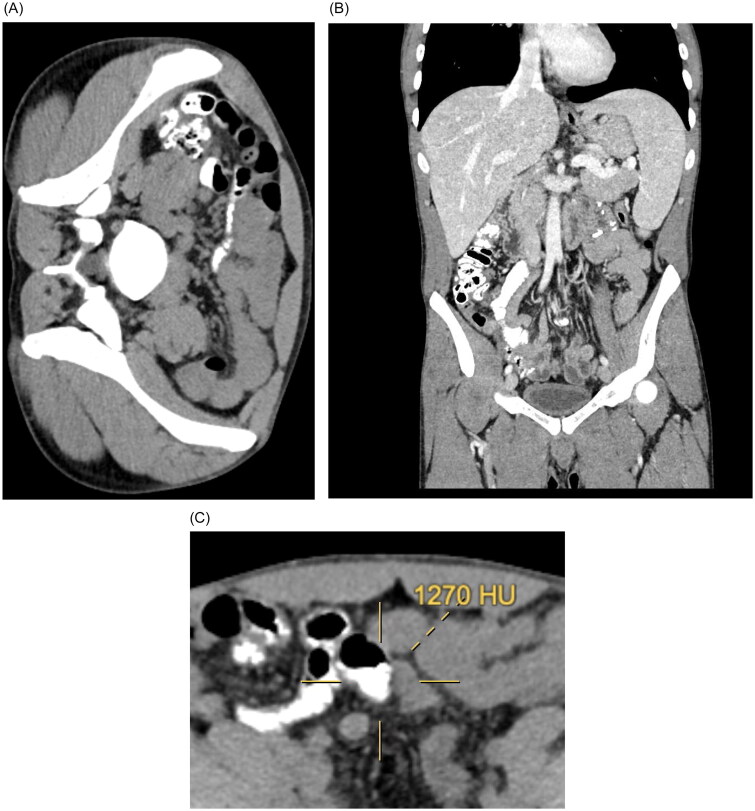
Computed Tomography (CT) Scan Demonstrating Radiopacity in the Bowel due to Sodium Zirconium Cyclosilicate (SZC). Panel A: Axial non-contrast CT image of the abdomen showing heterogeneous high-density material within the lumen of the small intestine, especially in the distal ileum. The material presents a radiopacity comparable to oral contrast medium but without prior administration. The image highlights the gravitational layering effect of the radiodense material. Panel B: Coronal non-contrast CT image of the abdomen and pelvis again demonstrating the high-density material distributed throughout the bowel lumen. Panel C: Highlight of the Hounsfield units (mean 1270 HU).

The observed radiopaque fluid level was caused by Sodium Zirconium Cyclosilicate, which the patient took orally 2 h before admission. The dosage was 10 g, and it was added to the daily medications.

## Discussion

The present experience suggests that the observed radiopacity is linked to the consumption of SZC, a standard treatment for hyperkalemia. This case was the object of debate between clinicians in our institution and, in particular, between radiologists since the literature lacks the argument. Radiologists should be aware of this distinct radiographic appearance to avoid misinterpretations or confusion with other medical conditions that should be added to radiopacity due to orally administered radiopaque contrast media, bismuth, barium, and other drugs that possess radiopaque properties (Table 1).

**Table 1.** List of differential diagnoses of heterogeneous high-density material within the bowel lumen on CT imaging.

Type of agent	Description	CT attenuation (H.U.)
Orally administered radiopaque contrast agents.	They are used in imaging procedures to enhance image contrast (e.g. Barium).	From 3066 to 23423
Bismuth subsalicylate ingestion (e.g. Pepto-Bismol)	This medication contains a radiopaque substance that can show up on imaging.	From 3500 to 5400
Ingestion of radiopaque drugs	Certain medications (e.g. Lanthanum carbonate (LC), Sodium Zirconium Cyclosilicate (SZC)) can appear on imaging due to their radiopaque properties.	LC: 2500; SZC from 900 to 1100 HU
Ingestion of radiopaque foreign bodies	Certain materials, such as certain metals or plastics, can appear on imaging if ingested.	Variable
Bezoar	A collection of undigested material that can sometimes collect in the gastrointestinal tract and may appear radiopaque.	From −300 to 100 HU
Fecal impaction	Hardened stool stuck in the rectum or lower colon due to chronic constipation.	From −150 to 100 HU
Gastrointestinal stromal tumors (GISTs)	These tumors can occasionally calcify and show up as high density on imaging.	Precontrast from 30 to 35 postcontrast: from 50 to 60
Gallstone ileus	A condition in which a gallstone passes into the bowel and causes a blockage. The stone could appear as high density on imaging.	Cholesterol stones: 28–98 HU; pigment stones: 90–120 HU
Swallowed dental materials	Some dental materials, such as amalgam or materials used in root canal treatment, can appear as radiopaque if accidentally swallowed.	Variable
Enterolith	These mineral concretions or “stones” form in the intestinal tract and can be radiopaque.	From 1000 to 1200
Pica (ingestion of non-nutritive substances like clay, soil, etc.)	Some substances ingested due to pica can appear radiopaque on imaging.	Variable
Calcified lymph nodes or tumors in the bowel wall	In rare instances, these may protrude into the bowel and mimic intraluminal high-density material.	Variable

HU: Hounsfield Unit.

There is only an existing case report that aligns with our findings regarding SZC’s radiopaque appearance. The distribution of the material observed on the X-ray indicates that it was ingested orally, with a greater concentration in the lower part of the small intestine and the descending and transverse parts of the large intestine. The material’s density is significantly higher than the surrounding intestine content, resembling oral contrast material, with a mean density of 1230 HU. However, since only SZC was ingested and no other contrast material or radiodense medication was administered, it is evident that the SZC is responsible for this effect. The radiopacity of the SZC, which contains zirconium, explains the observed phenomenon.

While the radiodensity did not impede the visibility of surrounding structures or lesions, it may impact the assessment of any intraluminal processes or masses. Additionally, no external issues were observed in the abdominal and pelvic regions. It is essential to note the potential impact of zirconium on image quality in other medical contexts, such as dental implants, as it may have relevance to this case. These findings are compatible with previous reports on titanium, zirconium, and binary titanium–zirconium composite dental implants and the artifacts in multimodal imaging found in patients possessing such implants [5], and on the impact of zirconium implants on image quality [6]. SZC is also a possible confounder when performing a bone mineral density (BMD) measurement using dual-energy X-ray absorptiometry (DEXA) [7].

In conclusion, SZC is radiopaque, and its ingestion can be a challenge in the interpretation of a radiological pattern of radiodense bowel. Communication between doctors and radiologists is crucial, particularly concerning patients’ medication histories. It is essential to inform the radiologist about using SZC before a CT scan, as this can prevent any misinterpretations, decrease the need for further testing, and enhance the accuracy of diagnoses.
